# Hydroa Vacciniforme: A Rare Pediatric Photodermatosis

**DOI:** 10.7759/cureus.82079

**Published:** 2025-04-11

**Authors:** Mouna Guechchati, Hanane Baybay, Meryem Zeryouhi, Layla Tahiri Elousrouti, FatimaZahra Mernissi

**Affiliations:** 1 Department of Dermatology, University Hospital Hassan II, Faculty of Medicine, Pharmacy and Dental Medicine, Sidi Mohamed Ben Abdellah University, Fez, MAR; 2 Department of Pathology, University Hospital Hassan II, Faculty of Medicine, Pharmacy and Dental Medicine, Sidi Mohamed Ben Abdellah University, Fez, MAR

**Keywords:** epstein-barr virus, hydroa vacciniforme, pediatric, photodermatosis, photosensitivity

## Abstract

Hydroa vacciniforme (HV) is a rare, chronic photodermatosis linked to Epstein-Barr virus (EBV) infection. We report a 12-year-old child with recurrent vesiculobullous facial lesions and photosensitivity for six years. Clinical examination revealed hemorrhagic crusts with atrophic scars. Dermoscopy showed whitish streaks and rosettes. Histopathology demonstrated epidermal spongiosis and a dense dermal lymphoid infiltrate. Immunohistochemistry confirmed CD3+, CD5+, CD7+, and CD8+ lymphocytes, with granzyme B and perforin expression, but no CD56. EBV serology was positive. Differential diagnoses, including cutaneous lupus, leishmaniasis, and porphyria, were excluded. The patient was managed with strict photoprotection, leading to sustained remission over two years. Early diagnosis and sun protection are essential for managing HV, as seen in our case.

## Introduction

Hydroa vacciniforme (HV) is a rare, idiopathic, and sporadic chronic photodermatosis characterized by recurrent vesicles and crusts on sun-exposed skin, typically leading to vacciniform or varioliform scarring [1]. It is a childhood photosensitivity disorder, initially considered benign due to its spontaneous resolution in adolescence [2]. Recent studies have linked HV to Epstein-Barr virus (EBV) infection. The role of EBV in HV pathogenesis remains unclear, but it is believed that EBV-induced immune dysregulation may play a role in disease development. Treatment options remain limited, with strict sun protection being the primary approach [3].

Although dermoscopy of hydroa vacciniforme is rarely discussed in the literature, it played a crucial role in this case by helping to exclude two major differential diagnoses, namely, lupus and leishmaniasis. The absence of dermoscopic signs characteristic of these diseases, such as the starburst pattern or corneal plugs, reinforced the clinical suspicion of hydroa vacciniforme. This case highlights the importance of a clinical, dermoscopic, and histological correlation to establish a rapid diagnosis, which can help minimize aesthetic damage by starting the appropriate treatment early.

## Case presentation

We report the case of a 12-year-old girl from a non-consanguineous marriage who had been experiencing recurrent fluid-filled lesions on the central face for six years, with periods of remission and exacerbation, as well as a history of photosensitivity. The evolution was typical, with vesicular lesions appearing predominantly after sun exposure confined to the photo-exposed areas of the central face.

**Figure 1:**
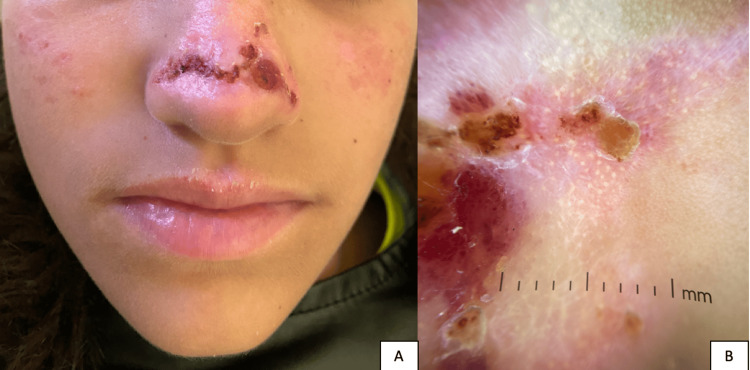
(A) Clinical presentation of hydroa vacciniforme with hemorrhagic crusts and atrophic varioliform scars on the cheeks and nasal tip. (B) Dermoscopy showing whitish scar-like areas scattered with rosettes and small, rounded follicular plugs.

Clinical examination revealed hemorrhagic crusts associated with atrophic and varioliform scars on the cheeks and nasal tip (Figure 1A). There was no mucosal involvement (oral, conjunctival, corneal, or genital).

The patient had no associated systemic symptoms such as joint swelling or pain, hair loss, lymphadenopathy, or hepatosplenomegaly. She also had no personal or family history of autoimmune disorders or similar dermatological conditions. There was no recent travel to a region endemic for leishmaniasis.

The dermoscopic image reveals whitish scar-like areas scattered with rosettes and small, rounded follicular plugs (Figure 1B). Unlike leishmaniasis, where facial follicular plugs often appear as peripheral teardrop-shaped structures, this pattern differs. It also contrasts with discoid lupus erythematosus, where follicular plugs are typically found at the periphery of scarring areas, indicating active disease. Additionally, the linear and dotted vascular pattern observed here differs from the short vessels seen in leishmaniasis and the arborizing vessels characteristic of discoid lupus. Also, lupus erythematosus and leishmaniasis were ruled out, as antinuclear antibodies and the leishmaniasis smear were negative. Laboratory studies, including complete blood count with differential, platelet count, liver and renal function tests, and red blood cell porphyrin levels, were all within normal limits.

Epstein-Barr virus (EBV) serology revealed positive IgG antibodies and negative IgM; however, PCR was not performed.

**Figure 2:**
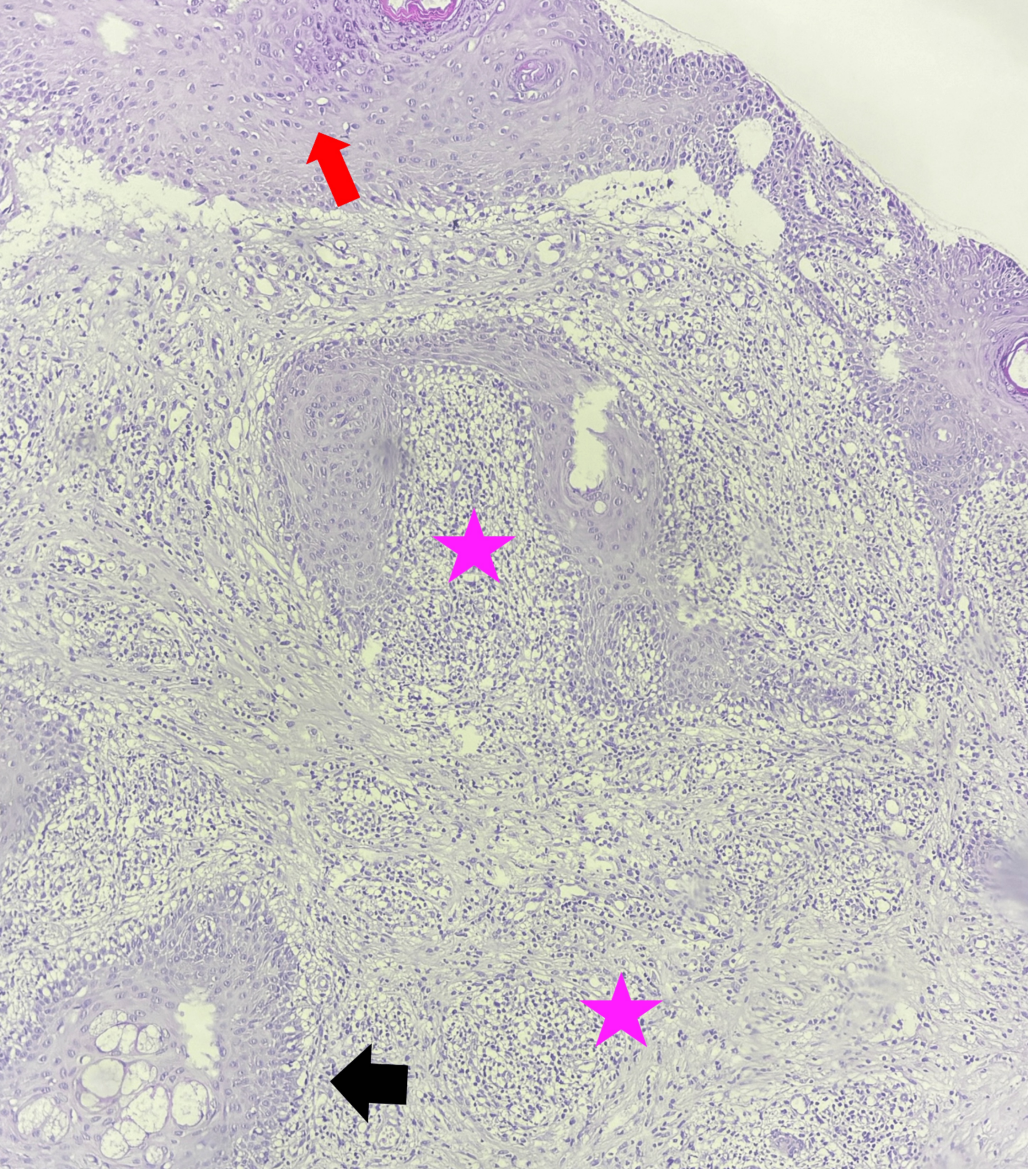
Histopathological findings of hydroa vacciniforme showing epidermal spongiosis (red arrow) with a dense dermal lymphoid infiltrate (pink star) located periadnexally and perivascularly (black arrow) (H&E stain, ×100 magnification). H&E: hematoxylin and eosin

A skin biopsy of the lesions demonstrated an acanthotic epidermis with overlying orthokeratotic hyperkeratosis and focal spongiosis. The dermis contains a dense inflammatory infiltrate composed of lymphocytes, located periadnexally and perivascularly (Figure 2).

**Figure 3:**
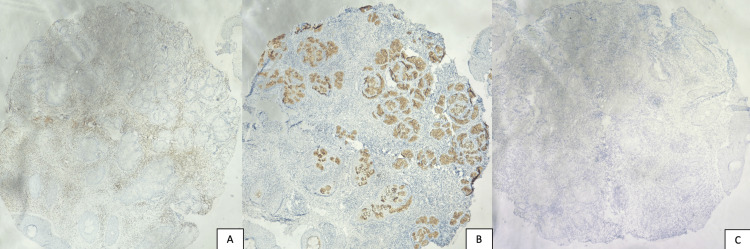
Immunohistochemical staining of the dermal lymphoid infiltrate: (A) CD3 expression highlighting T-cell predominance, (B) perforin positivity indicating cytotoxic activity, and (C) absence of CD56 expression, excluding NK/T-cell lymphoma.

In the immunohistochemical findings, the dermal lymphoid infiltrate, which showed positivity for CD3 (Figure 3A), CD5, CD7, and CD8, also included some cells that showed positivity for granzyme B and perforin (Figure 3B), indicating T-cell cytotoxic activity, with no CD56 expression (Figure 3C), helping to exclude NK/T-cell lymphoma. These findings supported a diagnosis of hydroa vacciniforme.

Unfortunately, due to limited financial resources, chromogenic in situ hybridization (CISH) analysis for EBV DNA could not be performed.

The patient initially received fusidic acid both topically and orally, along with strict photoprotection combining both chemical and physical measures. Chemical photoprotection consisted of the application of a broad-spectrum sunscreen (SPF 50) specifically formulated for pediatric use and suited to her skin type, reapplied every two hours. Physical photoprotection included behavioral measures such as wearing wide-brimmed hats and protective clothing and avoiding sun exposure during peak UV hours to minimize ultraviolet radiation exposure. She showed excellent improvement with a two-year follow-up with close monitoring.

## Discussion

Hydroa vacciniforme (HV) belongs to a rare and poorly described group of pediatric photodermatoses [4]. It mainly starts in childhood, frequently resolving by adolescence or young adulthood, with onset typically occurring between one and 11 years of age. It appears to have a slight male predominance. Its prevalence is 0.1-0.5 cases per 100,000 per year [5].

Hydroa vacciniforme (HV) is now recognized as a cutaneous manifestation of chronic active Epstein-Barr virus (EBV) infection, with lesions typically triggered by sun exposure [4]. The condition primarily affects sun-exposed areas such as the cheeks, nose, ears, and, less frequently, the forearms or hands. Clinically, the disease follows a chronic and recurrent course, with vesicles appearing shortly after sun exposure, subsequently evolving into crusts and leaving characteristic varioliform scars [6].

Although the disease is generally considered self-limiting and tends to resolve by adolescence, uncommon and more severe presentations have been reported. These include ocular involvement, ear and nasal deformities, or even finger contractures [2]. Importantly, the clinical presentation is the key factor in assessing prognosis. Features such as severe cutaneous involvement, loss of synchronization with sun exposure, lymphadenopathy, hepatosplenomegaly, or general deterioration should raise suspicion for a systemic form of chronic active EBV infection. This form carries a greater risk of progression to lymphoma and therefore warrants close, long-term monitoring [4].

The differential diagnosis of HV includes several blistering disorders triggered by light exposure, such as erythropoietic protoporphyria (EPP), bullous lupus erythematosus, solar urticaria, hydroa aestivale, and porphyria cutanea tarda (PCT). In our patient, a detailed clinical dermoscopical assessment, histopathological examination, and laboratory investigations allowed us to confirm the diagnosis of HV while ruling out other differential diagnoses [7].

A careful dermoscopic analysis can highlight key differences, aiding in the exclusion of major differential diagnoses. In this case, dermoscopy was instrumental in distinguishing HV from other conditions such as leishmaniasis and lupus erythematosus. The absence of characteristic dermoscopic signs, such as the peripheral teardrop-shaped follicular plugs seen in leishmaniasis or the active disease indicators such as peripheral follicular plugs in lupus [8], helped confirm the diagnosis of HV. Furthermore, the linear and dotted vascular pattern observed in our case contrasted sharply with the typical vascular patterns seen in both leishmaniasis and lupus, which further supported the diagnosis of hydroa vacciniforme. This underscores the importance of dermoscopy as a non-invasive tool in providing valuable diagnostic insights, particularly when dealing with difficult-to-diagnose cases and differentiating between similar-appearing disorders [9]. Table 1 highlights the distinguishing features between hydroa vacciniforme and its main differential diagnoses.

**Table 1:** Comparative features of HV, DLE, and cutaneous leishmaniasis. HV: hydroa vacciniforme, DLE: discoid lupus erythematosus, EBV: Epstein-Barr virus, CISH: chromogenic in situ hybridization

Feature	HV	DLE	Cutaneous leishmaniasis
Age/gender	Childhood, frequently resolving by adolescence	Typically young to middle-aged adults; more common in females	Any age; often in endemic areas
Trigger	Sun exposure (photosensitivity)	Sun exposure	Insect bites in endemic regions
Clinical lesions	Recurrent vesicles → crusts → varioliform scars on photo-exposed areas	Erythematous plaques with atrophic centers and adherent scales	Nodules/ulcers with raised borders in exposed areas
Common dermoscopic features	Whitish scar-like areas, rosettes, follicular plugs (our case)	Whitish scales, follicular plugs, arborizing blood vessels	Starburst pattern, yellow teardrop-shaped structures, central ulceration, hairpin, comma-shaped, vessels
Histopathology	Epidermal acanthosis, orthokeratotic hyperkeratosis, dermal lymphocytic infiltrate (perivascular and periadnexal)	Hyperkeratosis, follicular plugging, basal vacuolar degeneration, periadnexal/perivascular infiltrates	Mixed inflammatory infiltrate, amastigotes in macrophages (Leishman-Donovan bodies)
Immunohistochemistry	CD3+, CD5+, CD7+, CD8+, granzyme B+, perforin+, CD56-	Not routinely used; when done, interface T-cell infiltrate	Not routinely used; macrophage markers may be helpful
EBV findings	Positive EBV IgG; CISH testing for EBV DNA not performed	Not linked to EBV	Not linked to EBV
Systemic signs	None in our patient	May have systemic lupus signs in some cases	May have regional lymphadenopathy

Therefore, clinicians should suspect HV in children presenting with recurrent vesicular eruptions on sun-exposed areas that evolve into varioliform scars, particularly when there is a history of photosensitivity, especially in cases where there is a lack of characteristic dermoscopic findings of other pathologies.

In managing HV, various therapeutic options have been explored, although evidence remains limited due to the rarity of the condition and the absence of randomized trials [5]. Preventive measures remain the cornerstone of management. These include rigorous photoprotection through the use of broad-spectrum sunscreens (SPF 30 or preferably 50) with high UVA protection, applied generously 15-30 minutes before sun exposure and reapplied every two hours or after swimming or towel-drying [10]. Sunscreens remain the most effective therapeutic agents and are essential even when other treatments are used [6]. Physical photoprotection, such as wearing thick or specialized sun-protective clothing, wide-brimmed hats, and UV-protective sunglasses, along with avoiding sun exposure during peak hours (11 a.m. to 3 p.m.), is equally crucial [10]. In moderate to severe disease, narrowband UVB or PUVA phototherapy may offer clinical improvement if carefully administered to avoid disease flares [5]. In more refractory cases, systemic therapies such as antimalarial agents, cyclosporine, or azathioprine have been used, although their efficacy is variable and their use in children must be weighed against the risk of adverse effects [11]. β-carotene and dietary fish oils rich in omega-3 fatty acids have been reported with occasional benefit in isolated cases [5]. Ultimately, treatment should be tailored based on severity, the patient's quality of life, and risk-benefit considerations.

## Conclusions

In summary, we report a 12-year-old girl with the characteristic clinical and histopathological findings of HV, emphasizing the importance of clinically suspecting the diagnosis in typical presentations on photo-exposed areas and confirming it through histopathological examination. The use of dermoscopy, as demonstrated in this case, can help exclude other conditions such as lupus (absence of a starburst pattern) and leishmaniasis (absence of corneal plugs), further supporting a diagnosis of hydroa vacciniforme.

To our knowledge, dermoscopic features of HV have not been previously described in the literature, which underscores the need for further attention to their detailed characterization. In our patient, strict photoprotection has led to sustained remission, demonstrating the importance of early intervention in managing the disease.
